# Rapid Joint Formulation Between PICU and Forensic Services: Early European Lessons from a Sheffield Pilot

**DOI:** 10.1192/j.eurpsy.2026.11328

**Published:** 2026-07-31

**Authors:** K. Prakash

**Affiliations:** Psychiatric Intensive Care Unit, SHSC, Sheffield, United Kingdom

## Abstract

**Introduction:**

Transitions between Psychiatric Intensive Care Units (PICUs) and Forensic Mental Health Services remain one of the least integrated points in psychiatric care. Fragmented communication and sequential referrals often prolong admissions and delay recovery. In mid-2025 a short Sheffield pilot introduced an early joint formulation model—a single collaborative meeting within 72 hours of PICU admission—to test whether shared forensic input could shorten decisions and reduce restrictive practice.

**Objectives:**

To assess the feasibility and early outcomes of the ≤72-hour joint formulation model on decision-making timelines, restrictive interventions, and team confidence, and to evaluate its potential for replication across European psychiatric systems.

**Methods:**

Over a two-month period, consecutive PICU admissions meeting forensic-threshold criteria were reviewed through structured joint meetings between PICU and Forensic teams. A concise pathway checklist and shared risk summary replaced the traditional sequential referral process. Qualitative reflections from MDT members and quantitative indicators (time-to-decision, seclusion episodes, readmissions) were examined descriptively

**Results:**

Preliminary findings suggested approximately one-quarter faster forensic decisions and a notable reduction in seclusion events compared with the preceding two-month baseline. Staff feedback reflected higher confidence in proportionate risk management and less moral fatigue. Patients described the process as “fairer” and “easier to understand.” The pathway has since been adopted locally and presented as a template for cross-service replication.

**Conclusions:**

Early joint formulation between PICU and Forensic teams can transform a boundary into a bridge. The approach improved efficiency, reduced restrictive practice, and strengthened morale without additional resources. It directly supports the EPA’s Integrated Care Framework, offering a practical model adaptable across Europe.

This late-breaking abstract highlights a replicable European service innovation combining data and compassion. It advances EPA priorities of integration, safety, and human-centred psychiatry by demonstrating measurable improvement through an inexpensive, collaborative process. The model exemplifies how shared formulation within 72 hours can restore dignity, accelerate recovery, and unite teams around patient-centred risk understanding.

**Disclosure of Interest:**

None Declared

